# Assessment of adiposity distribution and its association with diabetes and insulin resistance: a population-based study

**DOI:** 10.1186/s13098-019-0450-x

**Published:** 2019-06-27

**Authors:** Kan Sun, Diaozhu Lin, Qiling Feng, Feng Li, Yiqin Qi, Wanting Feng, Chuan Yang, Li Yan, Meng Ren, Dan Liu

**Affiliations:** Department of Endocrinology, Sun Yat-sen Memorial Hospital, Sun Yat-sen University, 107 Yanjiang West Road, Guangzhou, 510120 People’s Republic of China

**Keywords:** Diabetes, Insulin resistance, Body adiposity parameters, Visceral adiposity index, Body adiposity index, Lipid accumulation product index

## Abstract

**Background:**

Rational measures in estimating adiposity distribution in diabetic patients has yet to be validated. This study aims to provide insight about the possible links between routinely available body adiposity parameters and the development of both diabetes and insulin resistance.

**Methods:**

We performed a population-based cross-sectional study in 9496 subjects aged 40 years or older. All of the body adiposity measures including body mass index (BMI), waist circumference (WC), waist-hip ratio (WHR), waist-height ratio (WHtR), visceral adiposity index (VAI), body adiposity index (BAI) and lipid accumulation product index (LAP) were separately evaluated according to standard measurement methods. Diabetes was diagnosed according to the American Diabetes Association 2010 criteria.

**Results:**

All tested body adiposity measurements were significantly associated with fasting plasma glucose (FPG), oral glucose tolerance test (OGTT) 2 h glucose, HbA1c and fasting insulin. Compared with other adiposity phenotypes, LAP have shown the relatively strongest while BAI have shown the relatively weakest association with increased odds of both diabetes and insulin resistance across all logistic regression models. Additionally, LAP provided the best discrimination accuracy for diabetes [area under the curve (AUC): 0.658 95% confidence intervals (CI) 0.645–0.671] and insulin resistance (AUC: 0.781 95% CI 0.771–0.792) when compared with other body adiposity parameters.

**Conclusions:**

The LAP index seems to be a better indicator than other adiposity measures tested in the study to evaluate the association of visceral fat mass with diabetes and insulin resistance, which should be given more consideration in the clinical practice.

## Introduction

Diabetes is a major risk factor for premature death in the general population and results in a huge social and economic burden on the health systems worldwide [1, 2]. Obesity is closely associated with a higher incidence of type 2 diabetes and involved in the development of diabetic complications [3]. Considering the important characteristic of excessive body fat accumulation, numerous anthropometric measurements and surrogate adipose indices are used in field settings to assess the risk of diabetes and insulin resistance [4, 5]. However, the rational and preferred method in estimating body adiposity in diabetes patients has yet to be validated.

Body mass index (BMI) is one of the most commonly used clinical measure to determine obesity in individuals. However, when using as a measurement of visceral and subcutaneous body fat distribution, the limitations of BMI is also well documented in various populations [6–8]. When comparing estimates of anthropometric measures with respect to their ability to predict the percentage of body fat, Schulze et al. [9] found that waist circumference (WC) in men was considerably better correlated with body fat than BMI.

Dual-energy X-ray absorptiometry (DXA) is consider as one of the best methods in quantifying individual adiposity. Recently, body adiposity index (BAI) was proposed as a useful measure for quantifying adiposity of individuals, which was validated with strong accuracy and correlation with DXA-derived body fat composition [10]. However, clinical utility of body composition using BAI is criticized in later confirmation studies as the relative accuracy of BAI in predicting percentage of body fat is strongly confounded by sex and the degree of obesity [11, 12].

The visceral adiposity index (VAI) is a valuable indicator of visceral adiposity and adipose tissue dysfunction [13]. Research in various populations reported that VAI is closely associated with many health-related outcomes such as insulin resistance and type 2 diabetes [14, 15]. However, in obese elderly women, variation of BAI, but not VAI, was positively associated with diabetes related inflammatory cytokines including tumor necrosis factor-α and interleukin-6 [16, 17].

As a new anthropometric measure of lipid over accumulation, the accurate predictive value of lipid accumulation product index (LAP) in insulin sensitivity, diabetes and metabolic syndrome is also documented [18–20]. In addition, LAP was strongly correlated with interleukin-6 levels and many other adipocytokines based on recent publications [21].

Distribution of body fat accumulation is of great important in the development of insulin resistance and diabetes. However, the inconclusive relationship of body adipose composition with diabetes might have negative effect when determining the prevention and treatment strategies for the disease. To date, previously existing investigations have not been implemented to make a systematic comparison on the associations of all these adipose indices with diabetes and insulin resistance. We have therefore aimed to determine a relatively superior body adiposity parameter associated with both diabetes and insulin resistance in a community-based population.

## Subjects and methods

### Study population and design

We performed a cross-sectional study in a community in Guangzhou, China from June to November 2011. The study population was from the REACTION study and details of this study have been published previously [22–24]. At first, 10,104 subjects aged 40 years or older were invited to participate by examination notices or home visits during the recruiting phase. After this, 9916 subjects signed the consent form and agreed to participate in the survey, including those with or without diabetes. Subjects who failed to provide information [WC: n = 186; hip circumference (HC): n = 42; height: n = 29; weight: n = 23; triglycerides (TG): n = 23; high-density lipoprotein cholesterol (HDL-C): n = 2; fasting plasma glucose (FPG): n = 15; oral glucose tolerance test (OGTT) 2 h glucose: n = 60; HbA1c: n = 40] were excluded from the analyses. Accordingly, a total of 9496 eligible individuals were included in the final data analyses. The study protocol was approved by the Institutional Review Board of the Sun Yat-sen Memorial Hospital affiliated to Sun Yat-sen University and was in accordance with the principle of the Helsinki Declaration II. Written informed consent was obtained from each participant before data collection.

### Clinical and biochemical data collection

Information on lifestyle factors, sociodemographic characteristics and family history were collected by using a standard questionnaire. Smoking or drinking habit was classified as ‘never’, ‘current’ (smoking or drinking regularly in the past 6 months) or ‘ever’ (cessation of smoking or drinking more than 6 months) [25]. A short form of the International Physical Activity Questionnaire (IPAQ) was used to estimate physical activity at leisure time by adding questions on frequency and duration of moderate or vigorous activities and walking [26]. Separate metabolic equivalent hours per week (MET-h/week) were calculated for evaluation of total physical activity.

Venous blood samples were collected for laboratory tests after an overnight fasting of at least 10 h. Measurements of FPG, fasting serum insulin, TG, total cholesterol (TC), HDL-C, low-density lipoprotein cholesterol (LDL-C), creatinine and γ-glutamyltransferase (γ-GGT) were done using an autoanalyser (Beckman CX-7 Biochemical Autoanalyser, Brea, CA, USA). HbA1c was assessed by high-performance liquid chromatography (BioRad, Hercules, CA). The abbreviated Modification of Diet in Renal Disease (MDRD) formula recalibrated for Chinese population was used to calculate estimated glomerular filtration rate (eGFR) expressed in mL/min per 1.73 m^2^ using a formula of eGFR = 175 × [serum creatinine × 0.011]^−1.234^ × [age]^−0.179^ × [0.79 if female], where serum creatinine was expressed as μmol/L [27].

### Anthropometric and body adiposity measurements

All participants completed anthropometrical measurements with the assistance of trained staff by using standard protocols. Body height and body weight were recorded to the nearest 0.1 cm and 0.1 kg while participants were wearing light indoor clothing without shoes. BMI was calculated as weight in kilograms divided by height in meters squared (kg/m^2^). WC was measured at the umbilical level with participant in standing position, at the end of gentle expiration. HC was measured the same way as WC, but the measuring tape was placed around the widest part of the buttocks. Waist-to-hip ratio (WHR) was calculated as WC divided by HC while waist-to-height ratio (WHtR) was calculated as WC divided by height in cm. VAI was determined by gender-specifc equations and calculated using the following formulas. Men: [WC/(39.68 + (1.88 × BMI))] × (TG/1.03) × (1.31/HDL-C); Women: [WC/(36.58 + (1.89 × BMI))] × (TG/0.81) × (1.52/HDL-C) [28]. WC was calculated in cm while HDL-C and TG in mmol/L. BAI was calculated using the equation as [(HC/(height^1.5^) − 18] [10]. HC and height were calculated in cm. LAP was calculated as (WC − 65) × TG in men and (WC − 58) × TG in women. WC was calculated in cm and TG in mmol/L [29, 30]. Three times consecutively blood pressure measurements were obtained by an automated electronic device in a 5-min interval by the same observer (OMRON, Omron Company, China). The average of three measurements of blood pressure was used for analysis.

### Definition of insulin resistance and diabetes

The insulin resistance index (homeostasis model assessment of insulin resistance, HOMA-IR) was calculated as fasting insulin (μIU/mL) × FPG (mmol/L)/22.5 [31]. Insulin resistance was defined by a HOMA-IR index within the top quartile (greater than 2.54) in the present study [32]. Diabetes was diagnosed according to the American Diabetes Association 2010 criteria, which included (1) FPG level of 7.0 mmol/L or greater, or (2) OGTT 2 h glucose level of 11.1 mmol/L or greater, or (3) HbA1c level of 6.5% or greater [33].

### Statistical analysis

Statistical analysis was performed using SAS version 9.2 (SAS Institute Inc, Cary, NC, USA). Continuous variables were presented as mean ± standard deviation (SD) except for skewed variables, which were presented as medians (interquartile ranges). Categorical variables were expressed as numbers (proportions). Differences between groups were performed by one-way ANOVAs. Comparisons between categorical variables were performed by the χ^2^ test. Physical activity levels, TG, FPG, OGTT 2 h glucose, HbA1c, fasting insulin, HOMA-IR, γ-GGT, WHR, WHtR, VAI, BAI and LAP were logarithmically transformed before analysis due to a non-normal distribution.

We primarily analyzed the associations of body fat content estimates (BMI, WC, WHR, WHtR, VAI, BAI and LAP) with FPG, OGTT 2 h glucose, HbA1c, fasting insulin and prevalence of insulin resistance and diabetes. Linear regression analyses were used to test for trends across groups. Pearson’s correlation and multiple regression analysis adjusted for age and sex were performed to test the correlations of body adiposity parameters with FPG, OGTT 2 h glucose, HbA1c and fasting insulin. The unadjusted and multivariate-adjusted logistic regression analysis was used to assess the risk of increased prevalent insulin resistance and diabetes in relation to 1-quartile increase in body adiposity measurements. Model 1 is unadjusted. Model 2 is adjusted for age. Model 3 is adjusted for age, sex, current smoking and drinking status, physical activity level, systolic blood pressure (SBP), LDL-C, γ-GGT, eGFR and antidiabetic treatment. By considering the variation in each quartile group, multivariate-adjusted logistic regression analysis was used to assess the prevalent diabetes and insulin resistance in relation to each quartile increase in body adiposity measurements. Odds ratios (OR) and the corresponding 95% confidence intervals (CI) were calculated. The discriminative ability of adiposity measurements on prevalent insulin resistance and diabetes was examined through area under the receiver operating characteristic curve (AUC) with 95% CI. Differences between the AUC of anthropometric measures were performed with a nonparametric approach [34]. To obtain a better assessment of the estimation power of the adipose distribution parameters, we used the optimal operating point with setting the minimum sensitivity of 70% for which we do not want sensitivity to fall below [35].

All statistical tests were two-sided, and a P value < 0.05 was considered statistically significant.

## Results

### Basic characteristics of the study population

The mean age was 55.9 ± 8.1 years among the 9496 enrolled individuals. Totally, 2054 subjects diagnosed with diabetes and the prevalence rate was 21.6% in this population. Accordingly, 606 (6.4%) subjects have been diagnosed with diabetes before the survey and among them 479 (5.0%) subjects were receiving antidiabetic treatment. Moreover, there were 1448 (16.3%) newly diagnosed diabetic patients based on the survey. The demographic and biochemical characteristics of the study population were shown in Table 1. Subjects in diabetes group differed from those in non-diabetes group in multiple clinical variables. All of the adiposity measurements in the study were significantly elevated in diabetes group (all P < 0.0001). Moreover, LAP was dramatically elevated in participants with diabetes in comparison with those without the disease [24.4 (14.4–41.2) vs 38.9 (22.5–61.9), P < 0.0001].

**Table 1:** Characteristics of study population

	Non-diabetes	Diabetes	P value
n (%)	7442 (78.4)	2054 (21.6)	< 0.0001
Age (years)	55.1 ± 7.7	58.8 ± 8.6	< 0.0001
Male [n (%)]	2062 (27.7)	630 (30.7)	0.008
Physical activity (MET-h/week)	22.4 (10.5–45.0)	21.0 (10.5–42.0)	0.497
Current smoking [n (%)]	731 (10.0)	204 (10.2)	0.817
Current drinking [n (%)]	238 (3.3)	75 (3.7)	0.311
SBP (mmHg)	124.4 ± 16.1	132.2 ± 16.8	< 0.0001
DBP (mmHg)	74.9 ± 9.8	76.9 ± 9.9	< 0.0001
TG (mmol/L)	1.21 (0.89–1.73)	1.57 (1.08–2.26)	< 0.0001
TC (mmol/L)	5.17 ± 1.22	5.28 ± 1.36	0.0006
HDL-C (mmol/L)	1.34 ± 0.36	1.23 ± 0.35	< 0.0001
LDL-C (mmol/L)	3.13 ± 0.94	3.18 ± 1.03	0.040
FPG (mmol/L)	5.29 (4.93–5.67)	6.46 (5.71–7.69)	< 0.0001
OGTT 2 h glucose (mmol/L)	6.90 (5.87–8.10)	11.67 (9.39–13.55)	< 0.0001
HbA1c (%)	5.80 (5.60–6.10)	6.60 (6.30–7.20)	< 0.0001
Fasting insulin (μIU/mL)	6.80 (5.10–9.40)	8.70 (6.00–12.40)	< 0.0001
HOMA-IR	1.60 (1.16–2.25)	2.61 (1.75–3.85)	< 0.0001
γ-GGT (U/L)	19.0 (14.0–27.0)	24.0 (17.0–36.0)	< 0.0001
eGFR (mL/min per 1.73 m^2^)	102.3 ± 22.6	99.5 ± 27.1	< 0.0001
BMI	23.4 ± 3.3	24.7 ± 3.5	< 0.0001
WC (cm)	80.7 ± 9.2	85.2 ± 9.9	< 0.0001
WHR	0.86 (0.82–0.90)	0.89 (0.85–0.94)	< 0.0001
WHtR	0.51 (0.47–0.54)	0.54 (0.50–0.58)	< 0.0001
VAI	1.55 (1.03–2.44)	2.20 (1.40–3.50)	< 0.0001
BAI	28.9 (26.3–31.7)	29.7 (26.8–32.9)	< 0.0001
LAP	24.4 (14.4–41.2)	38.9 (22.5–61.9)	< 0.0001

Data were mean ± SD or medians (interquartile ranges) for skewed variables or numbers (proportions) for categorical variables

P values were for the ANOVA or χ^2^ analyses across the groups

*MET-h/wk* separate metabolic equivalent hours per week, *SBP* systolic blood pressure, *DBP* diastolic blood pressure, *TG* triglycerides, *TC* total cholesterol, *HDL-C* high-density lipoprotein cholesterol, *LDL-C* low-density lipoprotein cholesterol, *FPG* fasting plasma glucose, *OGTT* oral glucose tolerance test, HOMA-IR homeostasis model assessment of insulin resistance, *eGFR* estimated glomerular filtration rate, *γ-GGT* γ-glutamyltransferase, *BMI* body mass index, *WC* waist circumference, *WHR* waist-hip ratio, *WHtR* waist-height ratio, *VAI* visceral adiposity index, *BAI* body adiposity index, *LAP* lipid accumulation product index

* P < 0.05 compared with non-diabetes group

### Body adiposity parameters and metabolic factors of glycometabolism

**Fig. 1:**
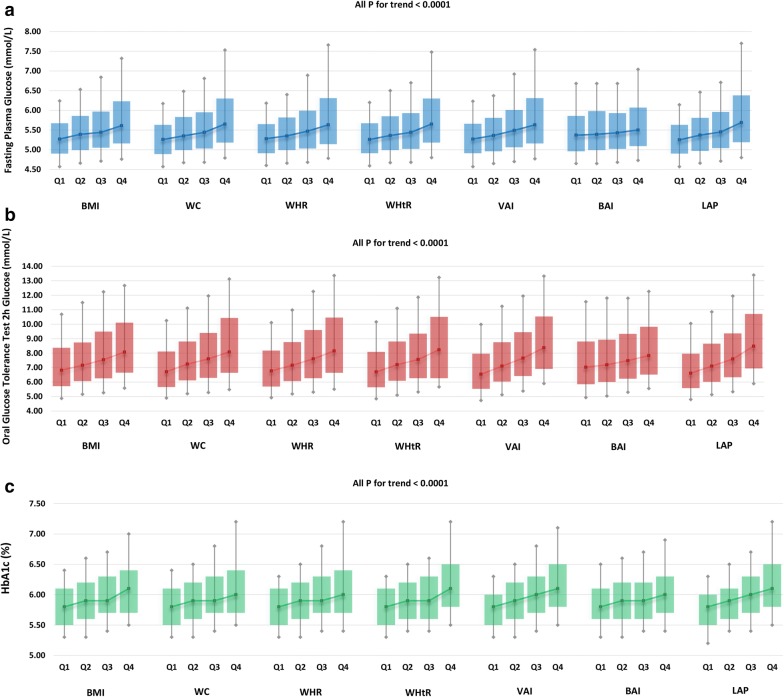
Distribution of FPG, OGTT 2 h glucose and HbA1c stratified by quartiles of body adiposity parameters **a** FPG, **b** OGTT 2 h glucose, **c** HbA1c. The box plot: upper horizontal line of box, 75th percentile; lower horizontal line of box, 25th percentile; solid point within box, 50th percentile (the median); upper solid point outside box, 90th percentile; lower solid point outside box, 10th percentile

Pearson’s correlation analysis revealed that body adiposity measurements including BMI, WC, WHR, WHtR, VAI, BAI and LAP were all significantly associated with increased FPG, OGTT 2 h glucose, HbA1c and fasting insulin (Table 2, all P < 0.0001). The associations were still persisted in multivariate linear regression analysis with age and sex adjusted (all P < 0.0001). Compared with other adiposity measurements, LAP reached the highest correlation coefficient with FPG (r = 0.22, P < 0.0001), OGTT 2 h glucose (r = 0.27, P < 0.0001), HbA1c (r = 0.24, P < 0.0001) and fasting insulin (r = 0.52, P < 0.0001). In multivariate linear regression analysis adjusted for age and sex, LAP was considerably most strongly correlated with HbA1c (β = 0.22, P < 0.0001) and fasting insulin (β = 0.52, P < 0.0001) while VAI was most strongly correlated with FPG (β = 0.21, P < 0.0001) and OGTT 2 h glucose (β = 0.26, P < 0.0001). However, as an independent determinant of body fat percentage, BAI was most weakly associated with FPG, OGTT 2 h glucose and HbA1c than other body adiposity measurements in both Pearson’s correlation and multivariate linear regression analysis. As shown in Fig. 1, with the elevating quartiles of adiposity phenotypes in the study, levels of FPG, OGTT 2 h glucose and HbA1c were all significantly increased (all P for trend < 0.0001).

**Table 2:** Pearson’s correlation and multiple regression analysis of body adiposity indexes associated with glucose metabolism indexes parameters

	FPG (mmol/L)				OGTT 2 h glucose (mmol/L)				HbA1c (%)				Fasting insulin			
	r	P value	St. β	P value	r	P value	St. β	P value	r	P value	St. β	P value	r	P value	St. β	P value
BMI	0.15	< 0.0001	0.15	< 0.0001	0.17	< 0.0001	0.17	< 0.0001	0.15	< 0.0001	0.15	< 0.0001	0.46	< 0.0001	0.46	< 0.0001
WC	0.19	< 0.0001	0.17	< 0.0001	0.20	< 0.0001	0.18	< 0.0001	0.19	< 0.0001	0.17	< 0.0001	0.45	< 0.0001	0.49	< 0.0001
WHR	0.18	< 0.0001	0.15	< 0.0001	0.20	< 0.0001	0.18	< 0.0001	0.19	< 0.0001	0.17	< 0.0001	0.28	< 0.0001	0.33	< 0.0001
WHtR	0.18	< 0.0001	0.16	< 0.0001	0.23	< 0.0001	0.20	< 0.0001	0.20	< 0.0001	0.17	< 0.0001	0.47	< 0.0001	0.47	< 0.0001
VAI	0.21	< 0.0001	0.21	< 0.0001	0.27	< 0.0001	0.26	< 0.0001	0.22	< 0.0001	0.21	< 0.0001	0.43	< 0.0001	0.42	< 0.0001
BAI	0.05	< 0.0001	0.09	< 0.0001	0.11	< 0.0001	0.13	< 0.0001	0.07	< 0.0001	0.09	< 0.0001	0.32	< 0.0001	0.35	< 0.0001
LAP	0.22	< 0.0001	0.20	< 0.0001	0.27	< 0.0001	0.25	< 0.0001	0.24	< 0.0001	0.22	< 0.0001	0.52	< 0.0001	0.52	< 0.0001

Multiple regression analysis is adjusted for age and sex

*BMI* body mass index, *WC* waist circumference, *WHR* waist-hip ratio, *WHtR* waist-height ratio, *VAI* visceral adiposity index, *BAI* body adiposity index, *LAP* lipid accumulation product index, *FPG* fasting plasma glucose, *OGTT* oral glucose tolerance test *r* correlation coefficient, *St. β* Standardized regression coefficient

### Body adiposity parameters with diabetes and insulin resistance

**Fig. 2:**
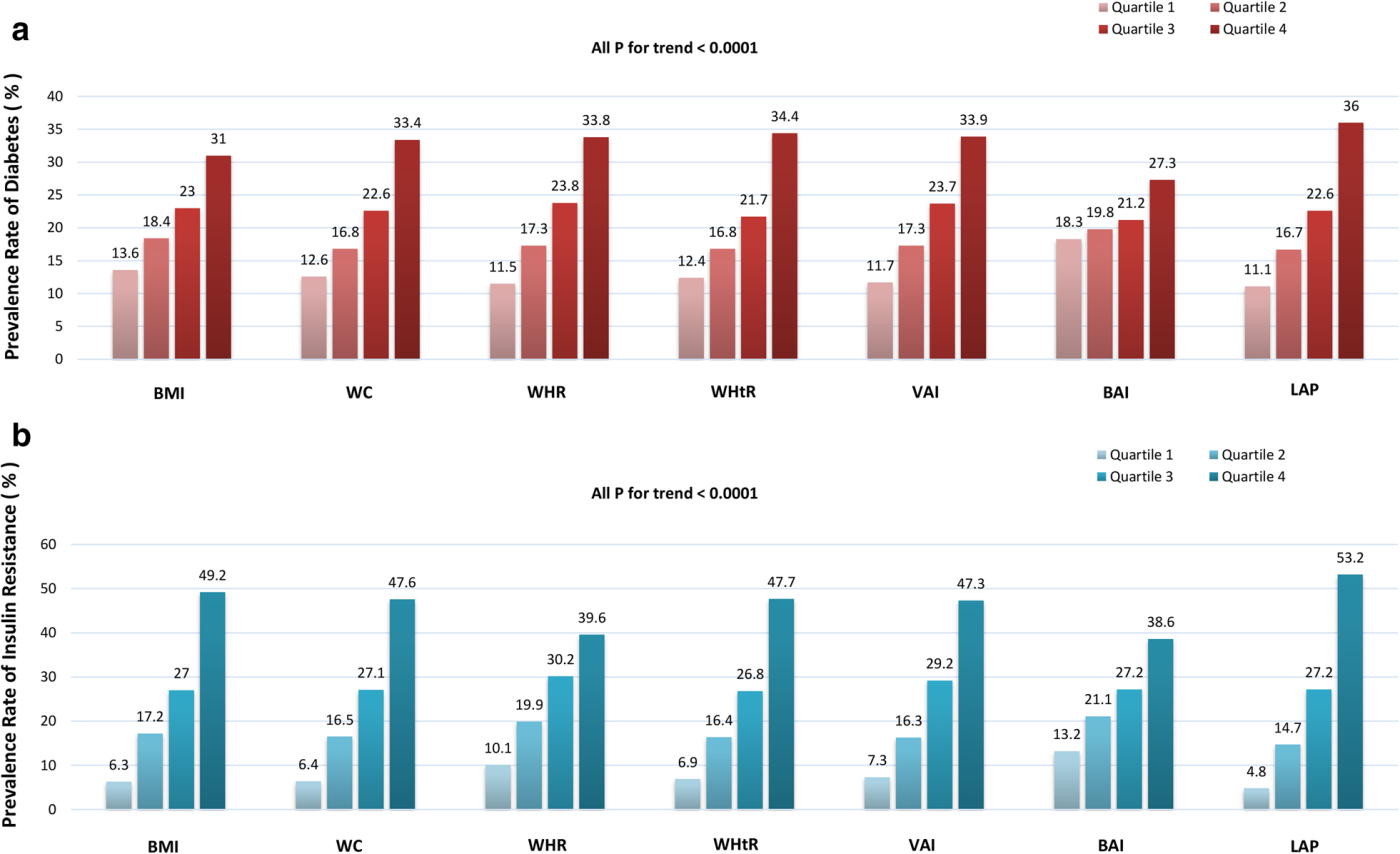
Prevalence of diabetes and insulin resistance in different quartiles of body adiposity parameters **a** diabetes, **b** insulin resistance

After this, we further studied the prevalence of diabetes and insulin resistance in different quartiles of body adiposity measurements. As showed in Fig. 2, the change in proportion of prevalent diabetes and insulin resistance were increased with the elevated quartiles of all tested adiposity parameters in the study (all P for trend < 0.001). The positive associations of BMI, WC, WHR, WHtR, VAI, BAI and LAP with diabetes and insulin resistance were consistently detected in both univariable and multivariable logistic regression analysis (Tables 3, 4). As shown in Table 4, compared with participants in quartile 1 of body adiposity measurements, multivariate-adjusted logistic regression analysis showed that participants in quartile 2, quartile 3 and quartile 4, respectively, have a significant correlation with increased odds of diabetes and insulin resistance. Compared with other adiposity phenotypes in the study, LAP have shown the relatively strongest while BAI have shown the relatively weakest associations with increased odds of both diabetes and insulin resistance across all logistic regression models. Further receiver operating characteristics curve represented consistent results and the LAP still performed the best discrimination accuracy for diabetes (AUC: 0.658 95% CI 0.645–0.671, all P < 0.05 except WHR) and insulin resistance (AUC: 0.781 95% CI 0.771–0.792, all P < 0.05) when compared with other adiposity phenotypes. We analysis the optimal operating point for the screening diabetes of the LAP. With a pre-assigned threshold of sensitivity for detecting a true-responder, greater than 70% in the present study, 25.5 of the LAP was thought to have a more appropriate screening effect for prevalent diabetes, of which the sensitivity and specificity were 70.0% and 52.0%, respectively. Accordingly, the sensitivity and specificity were 81.0% and 59.0% by using threshold 25.5 of the LAP for screening prevalent insulin resistance.

**Table 3:** Association of increased body adiposity parameters with risk of prevalent diabetes and insulin resistance

	1-Quartile change of increasing body adiposity parameters			AUC (95% CI)	P value
	Model 1	Model 2	Model 3		
Diabetes					
BMI	1.42 (1.36–1.49)	1.43 (1.37–1.50)	1.41 (1.33–1.49)	0.616 (0.602–0.630)	< 0.0001
WC	1.53 (1.46–1.60)	1.46 (1.39–1.54)	1.44 (1.35–1.52)	0.638 (0.624–0.651)	0.0003
WHR	1.57 (1.50–1.65)	1.47 (1.40–1.54)	1.44 (1.36–1.51)	0.648 (0.635–0.661)	0.140
WHtR	1.55 (1.48–1.63)	1.48 (1.41–1.55)	1.45 (1.37–1.54)	0.646 (0.632–0.659)	0.0276
VAI	1.57 (1.50–1.64)	1.54 (1.47–1.61)	1.49 (1.41–1.58)	0.645 (0.632–0.658)	0.0002
BAI	1.18 (1.13–1.24)	1.18 (1.13–1.24)	1.23 (1.15–1.30)	0.555 (0.540–0.569)	< 0.0001
LAP	1.65 (1.57–1.73)	1.60 (1.53–1.68)	1.60 (1.50–1.69)	0.658 (0.645–0.671)	–
Insulin resistance					
BMI	2.32 (2.20–2.44)	2.32 (2.21–2.44)	2.21 (2.09–2.33)	0.752 (0.741–0.763)	< 0.0001
WC	2.27 (2.16–2.39)	2.25 (2.14–2.37)	2.28 (2.15–2.41)	0.738 (0.727–0.749)	< 0.0001
WHR	1.74 (1.67–1.82)	1.73 (1.65–1.81)	1.73 (1.64–1.82)	0.678 (0.666–0.690)	< 0.0001
WHtR	2.26 (2.15–2.37)	2.24 (2.13–2.36)	2.11 (2.00–2.22)	0.744 (0.733–0.755)	< 0.0001
VAI	2.21 (2.11–2.32)	2.20 (2.09–2.31)	2.04 (1.93–2.15)	0.738 (0.726–0.749)	< 0.0001
BAI	1.58 (1.51–1.65)	1.58 (1.51–1.65)	1.65 (1.56–1.74)	0.648 (0.635–0.661)	< 0.0001
LAP	2.70 (2.56–2.85)	2.69 (2.55–2.84)	2.54 (2.40–2.69)	0.781 (0.771–0.792)	–

Data are odds ratios (95% confidence interval). Participants without diabetes or insulin resistance are defined as 0 and with diabetes or insulin resistance as 1

P value is calculated by the nonparametric approach to compare AUC of other anthropometric measures with the LAP group

Model 1 is unadjusted

Model 2 is adjusted for age

Model 3 is adjusted for age, sex, current smoking and drinking status, physical activity level, SBP, LDL-C, γ-GGT, eGFR and antidiabetic treatment

*BMI* body mass index, *WC* waist circumference, *WHR* waist-hip ratio, *WHtR* waist-height ratio, *VAI* visceral adiposity index, *BAI* body adiposity index, *LAP* lipid accumulation product index, *AUC* area under the receiver operating characteristic curve

**Table 4:** The risk of prevalent diabetes and insulin resistance according to quartiles of increased body adiposity parameters

	Quartile 1	Quartile 2	Quartile 3	Quartile 4
Diabetes				
BMI	1	1.58 (1.30–1.92)	1.96 (1.63–2.36)	2.92 (2.43–3.50)
WC	1	1.33 (1.09–1.63)	1.85 (1.53–2.24)	2.89 (2.39–3.50)
WHR	1	1.58 (1.30–1.93)	2.16 (1.77–2.63)	3.10 (2.55–3.77)
WHtR	1	1.39 (1.14–1.71)	1.80 (1.50–2.16)	3.05 (2.55–3.64)
VAI	1	1.50 (1.23–1.82)	2.13 (1.76–2.58)	3.34 (2.78–4.01)
BAI	1	1.25 (1.05–1.50)	1.37 (1.14–1.66)	1.88 (1.56–2.27)
LAP	1	1.58 (1.28–1.94)	2.27 (1.86–2.77)	4.06 (3.34–4.94)
Insulin resistance				
BMI	1	2.84 (2.31–3.49)	4.88 (4.01–5.95)	12.19 (10.04–14.79)
WC	1	2.72 (2.20–3.38)	5.11 (4.17–6.28)	12.96 (10.55–15.93)
WHR	1	2.11 (1.77–2.52)	3.68 (3.08–4.38)	5.58 (4.68–6.67)
WHtR	1	2.44 (1.99–3.00)	4.24 (3.52–5.11)	9.97 (8.29–11.99)
VAI	1	2.30 (1.89–2.80)	4.37 (3.62–5.26)	8.98 (7.48–10.79)
BAI	1	1.92 (1.62–2.27)	2.84 (2.39–3.38)	4.74 (3.98–5.66)
LAP	1	3.09 (2.45–3.90)	6.62 (5.30–8.27)	18.27 (14.66–22.77)

Data are odds ratios (95% confidence interval). Participants without diabetes or insulin resistance are defined as 0 and with diabetes or insulin resistance as 1

All models are adjusted for age, sex, current smoking and drinking status, physical activity level, SBP, LDL-C, γ-GGT, eGFR and antidiabetic treatment

*BMI* body mass index, *WC* waist circumference, *WHR* waist-hip ratio, *WHtR* waist-height ratio, *VAI* visceral adiposity index, *BAI* body adiposity index, *LAP* lipid accumulation product index

## Discussion

By selecting widely used clinical parameters of body adipose composition, this large-scale research in middle-aged and elderly Chinese suggests that elevated LAP has better relationship with both diabetes and insulin resistance than other adiposity measures tested in the study. Measurement of BAI, however, showed considerably weakest correlation with diabetes and insulin resistance when compared with other body adipose indexes. It is noteworthy that these conclusions highlight the clinical importance of considering LAP into the evaluation target for diabetes control, especially in overweight or obese adults.

A better understanding of the variation in distinct adiposity measures would probably shed light on the determination of obesity heterogeneity in subjects with diabetes. Despite BMI and WC are commonly measurements of individual obesity, utility of these parameters in predicting body fat is particularly inaccurate in distinguish between visceral adipose tissue and subcutaneous adiposity tissue. The increasing recognition of the differences in metabolic profiles between fat and fat-free mass have also stimulated interest in exploring simple and effective anthropometric indicators for evaluation of visceral adiposity. In the Dallas Heart study, WHR more accurate in estimating the risk of atherosclerosis than BMI and has been reported to be the best measurement of body adipose tissue mass [36, 37]. Nevertheless, results of the SAPHIR study stated that WHR was not inferior to BAI, BMI, or WHtR in its calculation of anthropometrical estimations, and the measurement of glucose homeostasis was hardly evaluated by WHR [38].

The BAI has emerged as the preferred parameter to estimates body composition as its correlation coefficient with DXA-derived percentage body fat was as high as 0.85 in Mexican–American and black subjects [10]. Recently, Alvim et al. [39] proposed that BAI is a better indicator for diabetes than BMI and WC in the Amerindian population. However, its predictive power for percentage body fat or diabetes in populations of other ethnicities seem to be inaccurate and inconsistent [9, 40]. The results of these studies, together with our present findings, suggested that BAI does not appear to be a prominent anthropometric indicator of glycometabolism disorder in Chinese population.

VAI is a novel indicator to assess both visceral fat distribution and adipose tissue dysfunction, which is reported to be closely correlated with risk of impaired glucose metabolism and diabetes [41, 42]. However, there are also studies suggested that VAI is unlikely to improve the prediction ability of type 2 diabetes beyond its components such as BMI and WC, and meanwhile, the performance of VAI for the diagnose of diabetes was not better than commonly available information on WHtR [43, 44]. In the present study, compared to other commonly available body adiposity indices such as BMI and WC, we found that VAI has a better in identification ability of prevalent diabetes. In addition, our results show that VAI is better than WHtR in the risk assessment for prevalent diabetes. Nevertheless, despite all strongly and independently related to glycometabolism parameters, the above measures involving WHtR, BMI and WC, have been shown to be superior to VAI in the association with insulin resistance based on current findings.

As a promising measurement of abdominal adiposity, LAP is first developed to reflect the combined anatomic and physiologic changes, which can accurately differentiate between visceral adiposity and subcutaneous adiposity by describing over accumulation of fat mass [45, 46]. The superiority of this adiposity assessment techniques is that a higher score will indicate a higher degree of lipid accumulation in the body. By secreting adipocytokines and increasing plasma concentration of free fatty acid, visceral fat is recently considered to be one of the multifunctional organ, which can interfere with the insulin signal and lead to insulin resistance and diabetes [47]. It has been found that individuals with greater degree of visceral fat also have greater degree of insulin resistance [48]. Besides accuracy, the ideal visceral adiposity parameter should be with simple calculation procedure. Actually, the process of LAP calculation is simple as (WC − 65) × TG in men and (WC − 58) × TG in women, which offers a better applicability in clinical practice. Therefore, LAP could reflect visceral adiposity accumulation and be used as a non-invasive assessment technique for assessing insulin resistance and diabetes.

Some limitations of the study should be discussed. First, no causal inference can be drawn due to the cross-sectional design of the current study. Further prospective cohort studies are needed to confirm the precise relationship between LAP and incidence of diabetes. Second, the imaging techniques such as DXA and magnetic resonance imaging are more accurate techniques for quantifying body fat distribution, but these examinations are costly and time consuming to be applied routinely in clinical settings. However, sampling analysis in assessing the concordance between generalizability of adiposity parameters using in this study and those reliable imaging techniques should be conducted to strengthen the findings of the present study. Third, findings of our study derived insight into the prominent role of LAP than other specific parameters of adipose tissue distribution in assessing diabetes and insulin resistance in subjects aged 40 years or older. In clinical settings, however, to improve the accuracy of estimating body adiposity, the LAP may also need to be firstly optimized to take account of age differences in body composition. Moreover, the study only including Chinese subjects and the results might not be representative of other ethnic groups, especially for those in the developed or undeveloped countries. To some extent, the population in the present study was still a convenience sample and selection bias is inevitable. Nevertheless, the present study, to our current knowledge, is the largest population-based study to explore the association of routine and non-traditional body adiposity indicators with both diabetes and insulin resistance in Chinese.

## Conclusion

Our findings add further evidence to understand the heterogeneity in the association of body adiposity compartments with prevalence of diabetes and insulin resistance. LAP may be suggested as an applicable parameter in assessing relationship between excess visceral fat mass and diabetes.

### Key messages

1. The present findings add further evidence to understand the heterogeneity in the association of body adiposity compartments with prevalent diabetes and insulin resistance.
2. The LAP index may be suggested as an applicable indicator in assessing relationship between excess visceral fat mass and diabetes.
3. The BAI does not appear to be a prominent anthropometric indicator of glycometabolism disorder in Chinese population based on the present findings.

## Data Availability

The work described was original research that has not been published previously, and not under consideration for publication elsewhere, in part or in whole. All authors believe that the manuscript represents valid work and have reviewed and approved the final version. Main document data and additional unpublished data from the study are available by sending Email to lizyhenu@163.com with proper purposes.

## Abbreviations

SBP: systolic blood pressure
DBP: diastolic blood pressure
TG: triglycerides
TC: total cholesterol
HDL-C: high-density lipoprotein cholesterol
LDL-C: low-density lipoprotein cholesterol
FPG: fasting plasma glucose
OGTT: oral glucose tolerance test
HOMA-IR: homeostasis model assessment of insulin resistance
eGFR: estimated glomerular filtration rate
γ-GGT: γ-glutamyltransferase
BMI: body mass index
WC: waist circumference
WHR: waist-hip ratio
WHtR: waist-height ratio
VAI: visceral adiposity index
BAI: body adiposity index
LAP: lipid accumulation product index
MET-h/wk: separate metabolic equivalent hours per week
